# The Congruency Sequence Effect 3.0: A Critical Test of Conflict Adaptation

**DOI:** 10.1371/journal.pone.0110462

**Published:** 2014-10-23

**Authors:** Wout Duthoo, Elger L. Abrahamse, Senne Braem, C. Nico Boehler, Wim Notebaert

**Affiliations:** Department of Experimental Psychology, Ghent University, Ghent, Belgium; University of Akron, United States of America

## Abstract

Over the last two decades, the congruency sequence effect (CSE) –the finding of a reduced congruency effect following incongruent trials in conflict tasks– has played a central role in advancing research on cognitive control. According to the influential conflict-monitoring account, the CSE reflects adjustments in selective attention that enhance task focus when needed, often termed conflict adaptation. However, this dominant interpretation of the CSE has been called into question by several alternative accounts that stress the role of episodic memory processes: feature binding and (stimulus-response) contingency learning. To evaluate the notion of conflict adaptation in accounting for the CSE, we construed versions of three widely used experimental paradigms (the colour-word Stroop, picture-word Stroop and flanker task) that effectively control for feature binding and contingency learning. Results revealed that a CSE can emerge in all three tasks. This strongly suggests a contribution of attentional control to the CSE and highlights the potential of these unprecedentedly clean paradigms for further examining cognitive control.

## Introduction

The last two decades have witnessed a remarkable increase in psychologists' interest in cognitive control – our ability to flexibly adjust to an ever-changing environment in order to pursue internal goals or to comply with external task demands. One crucial aspect of this flexibility lies in correcting our behaviour the moment it threatens to go wrong. According to the highly influential conflict-monitoring theory [1], the brain continuously monitors for such processing difficulties or *conflict* (see also [2], [3]). Following conflict detection, compensatory processes are triggered to optimize performance. Evidence for this conflict control loop has mainly been derived from so-called conflict tasks, such as the Stroop task, in which participants respond to the ink colour of word stimuli. More specifically, it has been systematically shown that congruency effects (the difference in performance between conflict-inducing incongruent and non-conflict congruent trials) are smaller following incongruent trials (e.g., the word ‘RED’ in green ink colour) than following congruent trials (e.g., ‘GREEN’ in green). This observation is referred to as the congruency sequence effect (CSE), and has been an essential asset to the conflict-monitoring theory, as it aligns with the notion of flexible, trial-to-trial conflict adaptation. Yet, the validity of interpreting this CSE in terms of conflict adaptation has been called into question by several alternative accounts that build on episodic memory processes. Given the wealth of behavioural and neuroscientific findings that have furthered theoretical insight into cognitive control based on this particular measure, it seems of cardinal importance to determine the specific relative contribution –if any– of selective attention to CSEs.

Since the first report of a CSE in the seminal paper by Gratton and colleagues [4], CSEs have become an increasingly popular measure to tap adjustments in attentional control (see [5], [6] for reviews). The effect proved to be highly robust, as it was replicated across various conflict tasks, such as the flanker [4], Simon [7], SNARC [8], and Stroop [9] tasks. Further research localized the underlying neural circuitry of conflict adaptation in medial and dorsolateral prefrontal cortex (see [10], for a review). However, a first wave of criticism drew researchers' attention to the effects of overlap between stimulus and response features across consecutive trials. Mayr, Awh, and Laurey [11], for instance, pointed out that complete stimulus repetitions evoke priming effects that mimic the typical pattern of CSEs. When exact repetitions were removed from the analysis, the congruency sequence effect was no longer found. A similar, but slightly more complex, alternative explanation can be derived from the feature integration account [12]. This account postulates that stimulus and response features are integrated into an event file. Responses are particularly slow when some aspects of an event are repeated but others are not. Hommel, Proctor, and Vu [13] indeed showed that partial stimulus feature repetitions slow down responding, whereas complete repetitions or complete alternations lead to fast reactions. Within this framework, there is no need for attention modulation– and thus no room for higher-order cognitive control processes.

A wealth of studies has since then been dedicated to systematically investigating the contribution of attentional adjustments to the CSE. In order to control for effects of feature integration, researchers expanded the stimulus set of their conflict tasks and restricted the analysis to a specific subset of trials in which feature overlap was absent [14]–[17] or kept equal [18]. Even though these studies generally confirmed a contribution of attentional control to the CSE, some studies failed to find any remaining sign of the CSE after controlling for feature binding effects [19], [20]. More recently, it was proposed to control for feature overlap *a priori*, by precluding such stimulus sequences. Duthoo and Notebaert [21] ran an 8-colour Stroop task devoid of feature overlap, providing strong evidence for a role of attentional control processes in bringing about the CSE. We refer to this version that is no longer confounded by feature binding as the *CSE 2.0*.

Still, this line of research elicited a second wave of criticism, which focused on employing stimulus designs of four or more stimuli while at the same time maintaining a 50% congruent/incongruent ratio. By doing so, specific stimuli that make up congruent trials occur more often than they would if features are selected randomly. As Mordkoff [22] has argued, increasing the proportion of congruent trials forces the irrelevant task dimension to become informative. According to the *contingency* account by Schmidt and colleagues [23], [24], such predictive relationships between stimuli and responses suffice to explain the CSE. In support of this claim, Mordkoff [22] as well as Schmidt and De Houwer [25] observed no remaining CSE in a design in which all contingencies were kept equal (see the Discussion section for further elaboration). This led Schmidt [23] to conclude that conflict adaptation might well be an illusion, and that the brain-behaviour correlations that are often cited in support of it might simply measure the memory biases that learning accounts have put forward. In the present paper, we construed versions of three major experimental conflict paradigms (colour-word Stroop, picture-word Stroop, and flanker tasks) in which, for the first time, *each* of the alternative episodic memory accounts are effectively controlled for. Importantly, we have set up these experiments so that all known confounds have been controlled for *a priori*, rather than *post hoc*. As such, we were able to critically test a contribution of selective attention to the CSE. To preview our conclusions, we found evidence for sequential modulation in all three tasks, confirming this *CSE 3.0* as an unprecedentedly clean measure for conflict adaptation.

## Experiment 1

Participants performed a series of three contingency- and feature-unbiased conflict tasks. They completed each of these tasks one after the other within a single experimental session. First, following Duthoo and Notebaert [21], we designed a vocal Stroop task with six colours in which feature repetitions were excluded *a priori*. However, we now also paired each word equally often with its congruent colour as with one of the remaining five incongruent colours. In this way, colour-word contingencies were rendered equal between congruent and incongruent trials, while the probability of congruent/incongruent trials was kept at 50%. Second, we also construed a picture-word interference task with 120 unique congruent and incongruent picture-word combinations, as to further minimize the contribution of episodic memory processes. Third, we further administered a flanker task that was pseudo-randomized along similar lines as the Stroop task described above, yet required manual responses. The order in which these three tasks were completed, was counterbalanced across participants. This procedure allowed us to generalize our findings across tasks, conflict types and response modalities, providing a strong first critical test of the CSE in the strict absence of episodic memory confounds.

### Method

#### Ethics Statement

The study was approved by the ethical committee of the Faculty of Psychology and Educational Sciences of Ghent University and in agreement with the Declaration of Helsinki.

#### Participants

A group of 24 Ghent University students (13 females; ages 17–23 years) provided written informed consent to participate in the experiment, lasting approximately 45 minutes. All participants had normal or corrected-to-normal vision, were not colour blind and reported to be skilled touch typists.

#### Stimuli and apparatus

A program written with T-Scope software [26] controlled stimulus presentation and response registration. Stimuli were displayed on a 17-inch monitor, with a viewing distance of approximately 50 centimetres. All text was presented in Courier, font size 22. Vocal responses were detected by means of a Sennheiser MD 421-U-4 microphone connected to an adapted voice key optimized for reaction time experiments [27]. Key presses were detected by means of a Dell QWERTY keyboard. For the Stroop task, stimuli consisted of six (Dutch) colour words printed in one of the six possible colours (red, green, blue, yellow, pink or brown) on a grey background. Participants had to react by saying the font colour out loud. For the picture-word interference task, stimuli consisted of pictures overlaid by a white text box in which a (Dutch) word was printed in black. Pictures were selected from the Severens, Van Lommel, Ratinckx, and Hartsuiker database [28] and paired with words such that there was no phonological or semantic relationship between them, and so that word length and the number of different responses elicited by the picture were as small as possible. The 240 picture-word combinations selected for the experiment are listed in Table S1. Just as in the Stroop task, participants had to react vocally, by saying the object shown on the picture out loud. For the flanker task, stimuli consisted of a five-letter string comprised of a target letter (S, D, F, J, K, or L) flanked on each side by two flanker letters, printed in black on a grey background. For the purpose of clarity, the target letter was underlined. Instead of responding vocally, participants had to react by pressing the keyboard key corresponding with the target letter.

#### Procedure and Design

Participants completed the Stroop, picture-word interference and flanker task in one session. They were randomly assigned to one of the six possible task sequence conditions, counterbalanced across participants. Each task consisted of five blocks of 49 trials, preceded by a short instruction slide and five practice trials. In between the experiments, as well as in between two blocks, participants were allowed a short, self-paced break. Speed and accuracy were equally stressed. No error feedback was provided. Below, the procedural details of each of the three tasks are summarized.

In the Stroop task, colour words were presented and participants were asked to name the font colour while ignoring the word's meaning. The relation between the word's meaning and its colour could either be congruent (e.g., ‘RED’ in red) or incongruent (e.g., ‘RED’ in green). Each word was paired equally often with its congruent colour and one of the remaining five incongruent colours. One such combination of word-colour pairings would consistently cross relevant and irrelevant features between incongruent trial pairs (e.g., ‘RED’ in green and ‘GREEN’ in red). As this would introduce unwanted contingencies, this stimulus set was not used. The remaining four possible sets of incongruent trials are listed in Table 1. Assignment of participants to stimulus sets was nested within the task sequence counterbalancing (so that the four participants in one order condition each responded to a different set of incongruent trials). The Stroop stimuli remained on screen until a response was recorded, with the maximum reaction time set to 2000 milliseconds. Once a response was registered by the microphone, a fixation cross replaced the stimulus and the experimenter coded the actual response given by the participant. The experimenter was blind to the congruency condition. After coding, the fixation cross remained on screen for another 1000 milliseconds before the next trial began.

**Table 1:** Four sets of unique incongruent colour-word pairings used in the Stroop task of Experiment 1.

Set 1		Set 2		Set 3		Set 4	
*Word*	*Colour*	*Word*	*Colour*	*Word*	*Colour*	*Word*	*Colour*
RED	blue	RED	green	RED	pink	RED	brown
BLUE	green	BLUE	yellow	BLUE	brown	BLUE	red
GREEN	yellow	GREEN	pink	GREEN	red	GREEN	blue
YELLOW	pink	YELLOW	brown	YELLOW	blue	YELLOW	green
PINK	brown	PINK	red	PINK	green	PINK	yellow
BROWN	red	BROWN	blue	BROWN	yellow	BROWN	pink

Participants were randomly assigned to one of the stimulus sets.

For each of the five blocks, the 49 stimuli were presented in pseudorandom sequences that obeyed to some specific constraints. The first trial, which was excluded from the statistical analysis, was always congruent. Of the remaining 48 stimuli, half were congruent (C) and half were incongruent (I). All 12 colour-word pairs were presented equally often. Taking into account the congruency level of the previous trial, each of the four possible sequences (CC, CI, IC, II) occurred with equal probability. Moreover, no more than four consecutive congruency level repetitions were allowed, as to avoid long runs of congruent or incongruent trials. Finally, complete stimulus repetitions or relevant or irrelevant feature repetitions were precluded, so that all stimulus and response features changed across two consecutive trials.

In the picture-word task, a compound picture-word stimulus was presented and participants were asked to name the picture while ignoring the word. The relation between the picture and the word could either be congruent (e.g., a picture of a cat with the word ‘CAT’ printed on top) or incongruent (e.g., a picture of a cat with the word ‘HOUSE’ on top). The timing and procedure were identical to that of the Stroop task. Each of the 120 pictures was coupled with its congruent word and one of the remaining 119 phonologically and semantically unrelated words. These 240 stimuli were divided into five sets of 48 trials (see Table S1 for a complete list). Each set contained 24 congruent and 24 incongruent unique pictures. Moreover, the distractor word of the 24 incongruent trials did not appear as a congruent picture-word combination within the same set. All participants completed these five sets of trials (one in each of the five blocks), yet the order in which they appeared was randomized. Within each block of 49 trials, the first trial, which was left out of the analysis, was an additional congruent picture-word combination. The remaining 48 trials were drawn out of one of the five stimulus sets, obeying to the following constraints: each of the four possible congruency level transitions was again presented equally often, and no more than four congruency level repetitions were allowed. The design guaranteed that within each block no stimulus or response feature was ever repeated.

In the flanker task, five-letter strings were presented and participants were asked to indicate the identity of the central target letter by pressing the corresponding keyboard key, while ignoring the flanking ones. Participants pressed the keyboard keys with the ring, middle, and index finger of their left and right hand. The rationale behind this particular finger-to-key mapping is that it is relatively well-learned, as most people have learned to touch type. The identity of the flankers could either be congruent (e.g., DDDDD) or incongruent (e.g., SSDSS). Each target letter was equally often flanked by a pair of congruent flankers and a pair of the remaining five incongruent flankers, creating four possible sets of incongruent trials (see Table 2). Timing was largely identical to the other two tasks, except that following the response, a fixation cross remained on screen for 1400 milliseconds before the next trial began (instead of the 1000 milliseconds used in the other tasks). This was changed in an attempt to keep the timing similar to that of the other tasks, since the flanker task did not require the experimenter to code the participants' responses. The randomization obeyed to the exact same constraints as in the Stroop task described above.

**Table 2:** Four sets of unique incongruent flanker-target pairings used in the flanker task of Experiment 1, 2A and 2B.

Set 1		Set 2		Set 3		Set 4	
*Flanker*	*Target*	*Flanker*	*Target*	*Flanker*	*Target*	*Flanker*	*Target*
SS	D	SS	F	SS	K	SS	L
DD	F	DD	J	DD	L	DD	S
FF	J	FF	K	FF	S	FF	D
JJ	K	JJ	L	JJ	D	JJ	F
KK	L	KK	S	KK	F	KK	J
LL	S	LL	D	LL	J	LL	K

Participants were randomly assigned to one of the stimulus sets.

### Results

Before being entered into the statistical analyses, the data were subjected to a trimming procedure (see below). Mean reaction times (RTs) and percentages of errors (PEs) were calculated for each cell of the design. Next, for each task we ran a mixed-design analysis of variance (ANOVA) with the within-subjects factors Previous Congruency and Current Congruency (two levels) and the between-subjects factor Task Order (six levels) on the mean RTs and PEs.

#### Stroop task

First, we removed all trials containing misses and false alarms caused by voice key malfunctioning (5.5%). For the reaction time analysis, error trials (1.8%), trials with RTs deviating more than 2.5 SD from the participant's grand mean (2.2%) and responses following an error trial or nonresponse (5.4%) were also excluded. Taken together, the data analysis was thus carried out on the remaining 85% of data.

**Figure 1:**
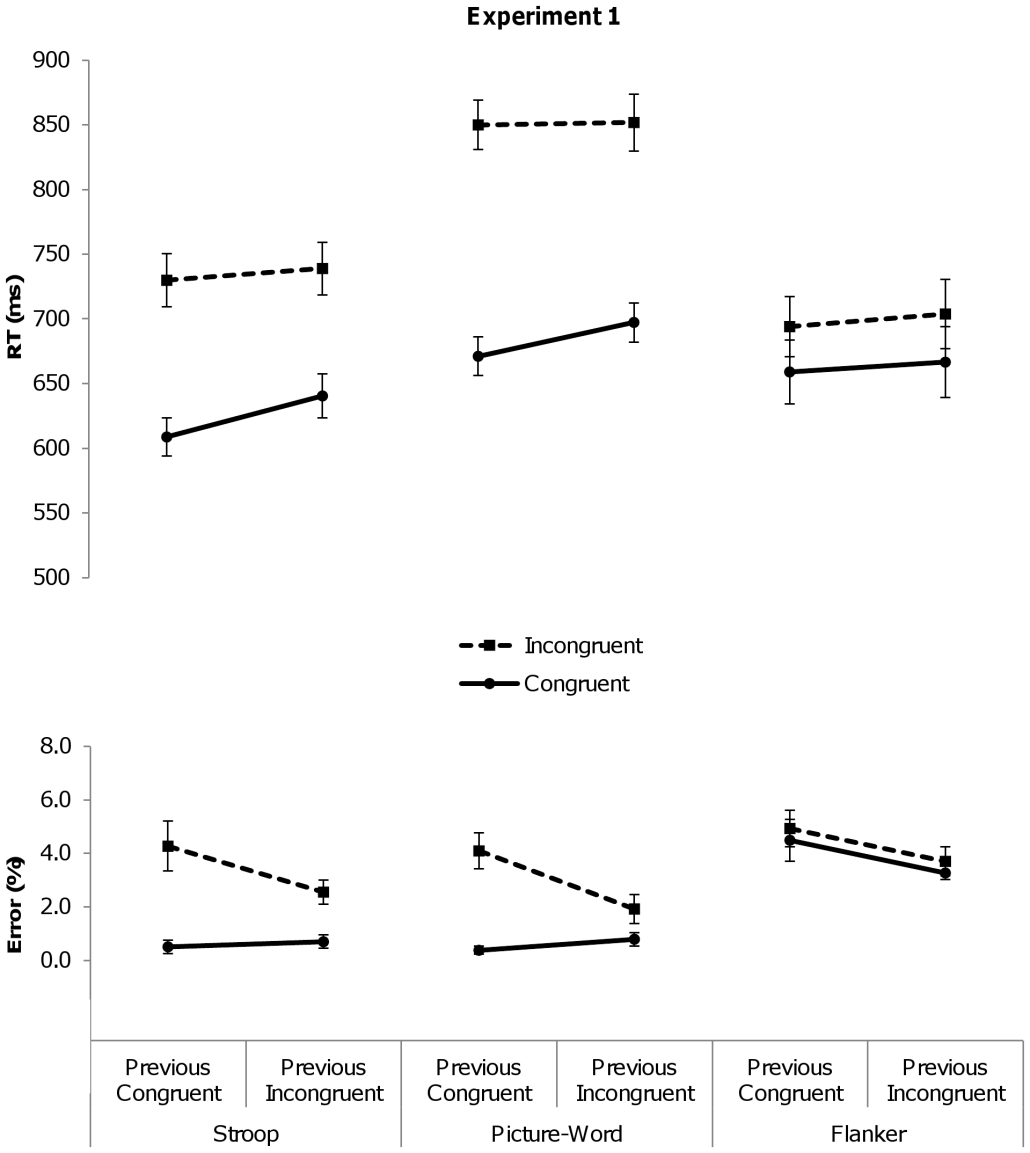
Mean reaction times (RTs, in milliseconds) and error percentages for congruent (solid line) and incongruent (dashed line) trials of Experiment 1 as a function of the congruency level of the previous trial, separately for the Stroop, picture-word and flanker task.

The RT analysis revealed a significant Stroop interference effect, *F*(1,18)  = 128.36, *p*<.001, *η^2^_partial_*  = .88: responses to congruent trials (M = 625 ms) were faster than responses to incongruent trials (M = 735 ms). The main effect of Previous Congruency also turned significant, *F*(1,18)  = 34.44, *p* = .001, *η^2^_partial_*  = .66, indicating that RTs were generally slower following incongruent trials (M = 690 ms) than following congruent trials (M = 669 ms). Importantly, the two-way interaction between Previous and Current Congruency was significant, *F*(1,18)  = 9.92, *p*<.01, *η^2^_partial_*  = .35, and did not vary with Task Order, *F*(5,18) <1, *ns*. As is depicted in Figure 1, the Stroop interference effect was smaller following incongruent (99 ms) than following congruent trials (121 ms), reflecting a CSE.

Overall accuracy was near ceiling (98%). The PE analysis revealed that incongruent trials evoked more erroneous responses (M = 3.4%) than congruent trials (M = 0.4%), reflected in a significant main effect of Current Congruency, *F*(1,18)  = 26.22, *p*<.001, *η^2^_partial_*  = .59. The main effect of Previous Congruency also turned significant, *F*(1,18)  = 4.64, *p*<.05, *η^2^_partial_*  = .21, indicating that accuracy was higher following incongruent trials (M = 1.6%) than following congruent trials (M = 2.4%). Importantly, the interaction between Previous and Current Congruency was significant, *F*(1,18)  = 4.60, *p*<.05, *η^2^_partial_*  = .20, and again did not vary with Task Order, *F*(5,18)  = 1.36, *p* = .29. As is depicted in Figure 1, the difference in error rates between congruent and incongruent trials was smaller following incongruent (1.9%) than following congruent trials (3.8%).

#### Picture-word interference task

We again removed all trials containing misses and false alarms caused by voice key malfunctioning (9%). For the reaction time analysis, error trials (1.6%), trials with RTs deviating more than 2.5 SD from a given participant's grand mean (2.4%) and responses following an error trial or nonresponse (8%) were also excluded. Taken together, the data analysis was thus carried out on the remaining 79% of data.

The RT analysis revealed a significant congruency effect, *F*(1,18)  = 265.18, *p*<.001, *η^2^_partial_*  = .94: responses to congruent trials (M = 684 ms) were faster than response to incongruent trials (M = 851 ms). The main effect of Previous Congruency also turned significant, *F*(1,18)  = 7.49, *p*<.05, *η^2^_partial_*  = .29, indicating that RTs were generally slower following incongruent trials (M = 775 ms) than following congruent trials (M = 761 ms). Importantly, the two-way interaction between Previous and Current Congruency was also significant, *F*(1,18)  = 7.97, *p*<.05, *η^2^_partial_*  = .31, and did not vary with Task Order, *F*(5,18) <1, *ns*. As is depicted in Figure 1, the congruency effect was significantly smaller following incongruent (155 ms) than following congruent trials (179 ms), reflecting a CSE.

Overall accuracy was near ceiling (98%). The PE analysis revealed that incongruent trials evoked more erroneous responses (M = 3%) than congruent trials (M = .6%), reflected in a significant main effect of Current Congruency, *F*(1,18)  = 24.5, *p*<.001, *η^2^_partial_*  = .58. The main effect of Previous Congruency did not reach significance, *F*(1,18)  = 3.99, *p* = .061. Importantly, the two-way interaction between Previous and Current Congruency was significant, *F*(1, 18)  = 11.19, *p*<.01, *η^2^_partial_*_  = _.38, and again did not vary with Task Order, *F*(5,18)  = 1.36, *p* = .29. As is depicted in Figure 1, the difference in error rates between congruent and incongruent trials was significantly smaller following incongruent (1.1%) than following congruent trials (3.7%), reflecting a CSE.

#### Flanker task

First, we removed all trials in which participants failed to respond before the response deadline (2.1%). For the reaction time analysis, error trials (4%), trials with RTs deviating more than 2.5 SD from the participant's grand mean (2.5%) and responses following an error trial or nonresponse (5.4%) were also excluded. Taken together, the data analysis was thus carried out on the remaining 86% of data.

The RT analysis only revealed a significant flanker interference effect, *F*(1,18)  = 78.01, *p*<.001, *η^2^_partial_*  = .81: responses to congruent trials (M = 663 ms) were faster than responses to incongruent trials (M = 699 ms). Unlike for the other conflict tasks above, the interaction between Previous and Current Congruency was not significant, *F*(1,18) <1, *ns*, indicating the absence of a CSE in the flanker task, irrespective of Task Order, *F*(5,18) <1, *ns*. As is depicted in Figure 1, the size of the flanker effect was of similar size following congruent (35 ms) as following incongruent (37 ms) trials.

Overall accuracy was near ceiling (96%). The PE analysis revealed a borderline significant main effect of Previous Congruency, *F*(1,18)  = 4.33, *p* = .052, yet no significant main effect of Current Congruency, *F*(1,18) <1, *ns*, indicating that incongruent trials did not evoke more erroneous responses (M = 4.3%) than congruent trials (M = 3.9%). Unlike for the other conflict tasks above, the interaction between Previous and Current Congruency was not significant, *F*(1,18) <1, *ns*, irrespective of Task Order, *F*(5,18) <1, *ns*. As is visualized in Figure 1, no CSE was found.

In order to test the hypothesis that the CSE was only present at the beginning of the task and dissipated [29], we have split the data into two halves (i.e., halfway the third block) and reran the analysis with the extra within-subjects factor Half (first or second). Results indicated that the CSE was absent in both the first and the second half (i.e., the three-way interaction between Half, Previous Congruency and Current Congruency was nonsignificant, *F*(1,23) <1, *ns*). In a next analysis, we also checked whether the order of the three experiments might have exerted an impact on the CSE in the flanker task, and, more specifically, whether the subset of participants who performed the flanker task first (*n* = 8) displayed a CSE. However, results showed that also in this subgroup, there was no sign of the CSE (*F*(1,7) <1, *ns*). We thank an anonymous reviewer for this suggestion.

## Experiments 2A and 2B

In Experiment 1, we showed that a CSE can still emerge once all known episodic memory confounds have been controlled for, providing strong support for a role of conflict adaptation. Both in the Stroop and picture-word interference task, we found reduced congruency effects following incongruent trials. In the flanker task, however, such sequential modulation was absent. Given this surprising deviation from the other results, and the general limitation of null findings, we decided to run two additional groups of participants: One group (Experiment 2A) performed the same flanker task as in Experiment 1, while another group (Experiment 2B) performed a flanker task that differed from Experiment 1 (and 2A) in three aspects: the maximum response time was reduced to 1200 milliseconds, the flanker letters preceded the target letter by 250 milliseconds, and the complete stimulus array remained on screen for only 400 milliseconds (thereby increasing the overall level of conflict; see e.g. [20], [30]). In the method section below, only changes relative to the design of the flanker task in Experiment 1 are listed, with everything else remaining the same.

### Method

#### Ethics Statement

The study was approved by the ethical committee of the Faculty of Psychology and Educational Sciences of Ghent University and in agreement with the Declaration of Helsinki.

#### Participants

Two groups of 24 Ghent University students (Experiment 2A: 18 females, ages 18–30 years; Experiment 2B: 22 females, ages 17–27 years) provided written informed consent to participate. Both lasted approximately 15 minutes. All participants had normal or corrected-to-normal vision and reported to be skilled touch typists.

#### Procedure and Design

Experiment 2A was an exact replication of the flanker task that was used in Experiment 1. For Experiment 2B, the same randomization and stimuli were used, but the presentation of these stimuli differed in three aspects: flanker letters preceded the target letter by 250 milliseconds instead of being presented simultaneously, and the complete stimulus array did not remain on screen until a response was recorded, yet disappeared after 400 milliseconds. Finally, the maximum response time was set to 1200 milliseconds.

### Results

First, we removed all trials in which participants failed to respond before the response deadline (Exp 2A:.4%; Exp 2B: 2.9%). For the reaction time analysis, error trials (Exp 2A: 5.9%; Exp 2B: 10.7%), trials with RTs deviating more than 2.5 SD from the participant's grand mean (Exp 2A: 2.2%; Exp 2B: 1.7%) and responses following an error trial or nonresponse (Exp 2A: 5.7%; Exp 2B: 10.9%) were also excluded. Taken together, the data analysis was thus carried out on the remaining 86% of data in Experiment 2A, and the remaining 74% in Experiment 2B.

**Figure 2:**
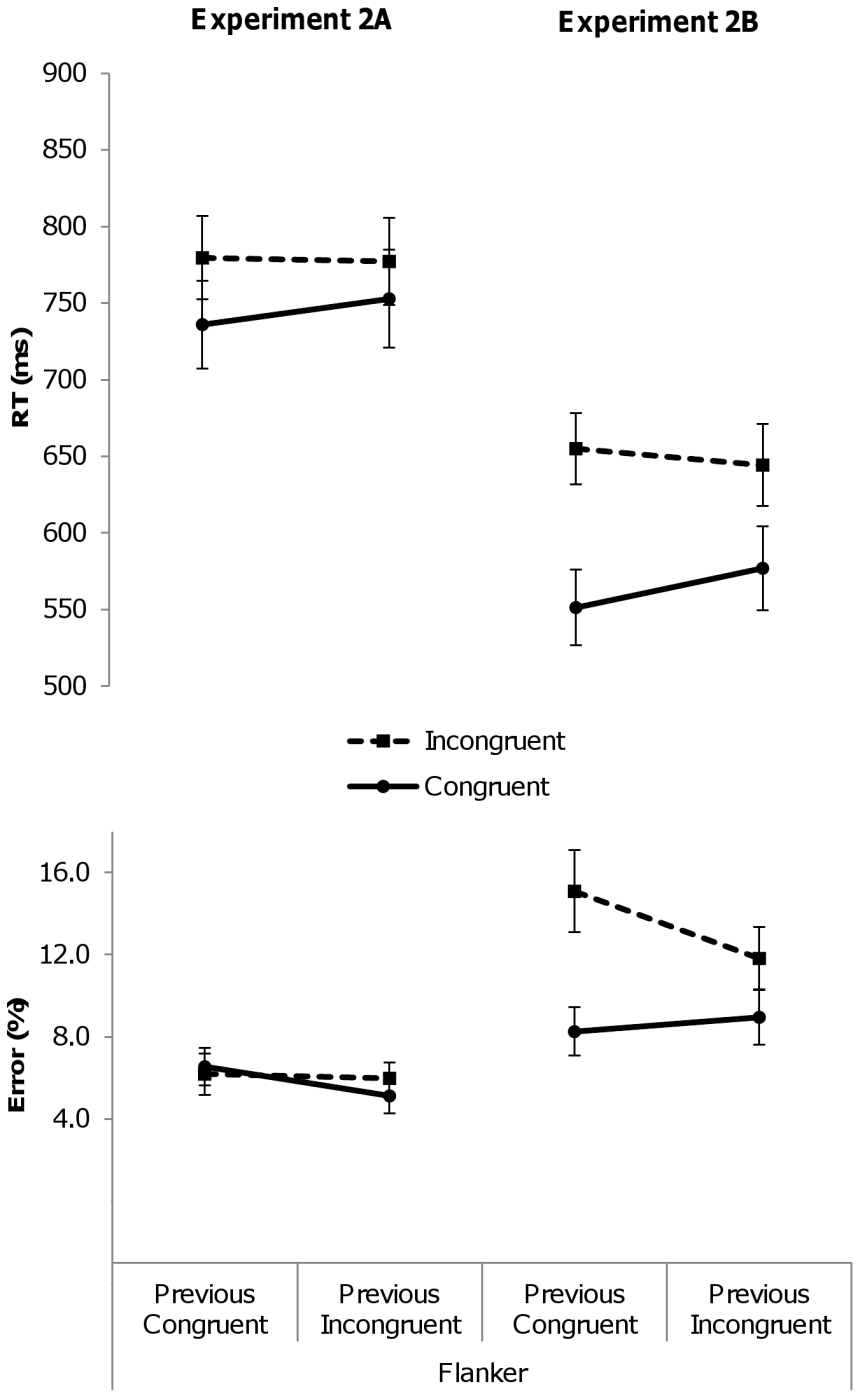
Mean reaction times (RTs, in milliseconds) and error percentages for congruent (solid line) and incongruent (dashed line) trials of the flanker task in Experiment 2A (left) and Experiment 2B (right) as a function of the congruency level of the previous trial. Error bars represent 95% confidence intervals around the mean.

The RT analysis revealed a significant flanker interference effect in both Experiment 2A, *F*(1,23)  = 42.29, *p*<.001, *η^2^_partial_*  = .65, and Experiment 2B, *F*(1,23)  = 83.48, *p*<.001, *η^2^_partial_*  = .78: responses to congruent trials (Exp 2A: M = 744 ms; Exp 2B: M = 564 ms) were faster than responses to incongruent trials (Exp 2A: M = 778 ms; Exp 2B: 649 ms). The main effect of Previous Congruency was not significant in Experiment 2A, *F*(1,23)  = 2.18, *p* = .15, nor in Experiment 2B, *F*(1,23)  = 2.27, *p* = .15. Unlike the previous flanker experiment, the interaction between Previous and Current Congruency reached significance this time, both in Experiment 2A, *F*(1,23)  = 4.41, *p*<.05, *η^2^_partial_*  = .16, and in Experiment 2B, *F*(1,23)  = 30.99, *p*<.001, *η^2^_partial_*  = .57. As is depicted in Figure 2, the size of the flanker effect was smaller following incongruent (Exp 2A: 24 ms; Exp 2B: 67 ms) compared to following congruent (Exp 2A: 44 ms; Exp 2B: 104 ms) trials, indicating a CSE.

In Experiment 2A, overall accuracy was high (94%). The PE analysis revealed neither a significant main effect of Current Congruency, *F*(1,23) <1, *ns*, indicating that incongruent trials did not evoke more erroneous responses (M = 6.1%) than congruent trials (M = 5.9%), nor a significant main effect of Previous Congruency, *F*(1,23) <1, *ns,* nor an interaction between Previous and Current Congruency, *F*(1,23) <1, *ns*. As is visualized in Figure 2, no sign of a CSE was found. In Experiment 2B, the analysis revealed a main effect of Congruency, *F*(1,23)  = 7.97, *p*<.05, *η^2^_partial_*  = .26: participants made fewer errors on congruent trials (M = 8.6%) than on incongruent trials (M = 13.5%). The main effect of Previous Congruency was not significant, *F*(1,23)  = 127, *p* = .27. In Experiment 2B, however, the interaction between Previous and Current Congruency almost reached significance, *F*(1,23)  = 4.27, *p* = .0503, *η^2^_partial_*  = .16. As is depicted in Figure 2, the size of the error flanker effect was smaller following incongruent trials (M = 2.9%) as compared to following congruent trials (M = 6.8%).

## General Discussion

In the present paper, we report strong evidence for a CSE in three adapted conflict paradigms that, for the first time, effectively controlled for each of the episodic memory confounds reported in the literature. Given the wealth of studies that have relied on the CSE to inspire, advance and frame further theorizing about cognitive control (see [5], [6] for reviews) and to gain insights into clinical pathologies like depression [31] and Parkinson's disease [32], this is an important empirical observation. Moreover, the paradigms presented here constitute a definite improvement over the highly prevalent yet contingency-biased four-alternative conflict tasks (i.e., in which the proportion of congruent trials is artificially increased from 25% to 50%; see [22]) and might therefore serve as a more viable tool to uncover the underlying neural circuitry of the control adjustments captured in the CSE.

These findings stand in apparent contrast to previous results. Mordkoff [22] as well as Schmidt and De Houwer [33] observed no remaining CSE in their contingency-unbiased Simon or Stroop task, respectively. However, some methodological differences between these and our designs might account for this discrepancy. First of all, the latter studies employed a four-alternative conflict task and chose to select features randomly, resulting in an experiment in which each word was equally predictive of its congruent and incongruent colours, but 75% of all trials were incongruent (compared to 50% in our designs). Introducing such proportion-congruency manipulation might have evoked a more sustained control mode, potentially obscuring the transient control adjustments reflected in the CSE. Second, Mordkoff [22] as well as Schmidt and De Houwer [25] controlled for feature integration and priming effects by restricting the analysis to a subset of specific trial transitions *post hoc*. Just as in our previous work [21], [34], we opted to leave out trials with feature repetitions prior to testing, by putting restrictions on the randomization. In doing so, the decision on the presence or absence of CSEs no longer relies on the analysis of a very limited and thus special subset of trials, but will, in principle, take into account all trials. For these reasons, we feel that the adapted conflict tasks employed here were better suited to identify a ‘clean’ CSE.

More importantly, yet, precluding contingencies from the start may be crucial in allowing conflict adaptation processes to emerge: Bugg [35] recently showed that conflict adaptation may be envisaged as a sort of “last resort that is engaged when reliance on one's environment, and in particular associative responding, is unproductive for achieving task goals” (p. 1). In other words, it might be the case that inserting contingencies and/or stimulus feature overlap in the design precludes the need to engage in potentially more metabolically costly attentional control adjustments. In such cases, simply picking up and adapting to these statistical regularities would be advantageous. However, it remains a challenge for future research to pinpoint the portion of the CSE that can be explained by executive control relative to episodic memory, as well as how exactly these influences interact in different experimental conditions. Yet, for the present purposes, we deem it important to show that the CSE was still found once episodic memory confounds were controlled for. The designs described in this paper might thus serve as a fruitful tool for tackling the challenges raised above.

Strikingly, the CSE in the flanker task appeared less robust than in the other two newly designed contingency-unbiased conflict tasks. In Experiment 1, in which participants completed the three tasks in randomized order, no sign of a flanker CSE was found. Nieuwenhuis and colleagues [20] reported similar null effects in a series of five experiments with an arrow version of the flanker task, when restricting their analysis to response changes (which, in our experiment, was the case for all trial transitions). Still, in Experiment 2A, the exact same task was run on a new group of participants, which evoked a similar conflict adaptation pattern as the Stroop and picture-word interference tasks, albeit only in RTs. The reason for this discrepancy between tasks might lie in the fact that flanker performance relied on manual responses that are inherently less intuitive than the simple naming instruction of the other two tasks and thus contained more individual variability, even though participants reported to be highly skilled touch typists. The higher error rates in the flanker task and lack of congruency effect therein indeed suggest that the letter-to-key mapping was maybe not as readily available as we assumed. This might render the paradigm more brittle, and may therefore call for the inclusion of more trials or more practice. Still, Experiment 2A clearly highlights that *real* conflict adaptation effects can be obtained with this task. Moreover, Experiment 2B provided strong additional support for the existence of a CSE in our flanker task set-up. Here, conflict was increased by presenting the flanker stimuli before the target letter (see also [30]) and shortening the presentation time. Results revealed a clear conflict adaptation pattern in reaction times as well as in accuracy. Taken together, we thus believe it is likely that the failure to obtain a reliable flanker CSE in Experiment 1 was due to a type-2 error.

In our experiments, the CSE was (mainly) driven by the relative slowing down and speeding up of congruent trials. It is indeed currently debated what the exact mechanisms are that drive the adaptation effects, and to what extent both congruent and incongruent trials modulate behaviour. In this respect, Schlaghecken and Martini [36] suggested that context (rather than conflict) is the crucial factor. Still, the relative cost induced by incongruent trials (as compared to congruent trials) was significantly reduced following conflict, both in terms of reaction times and accuracy. This suggests increased task focus following conflict. Moreover, determining the precise mechanisms at play by exploring aftereffects of congruent versus incongruent trials is problematic to begin with. As shown by Verguts, Notebaert, Kunde and Wühr [37], there are additional post-conflict slowing processes at play for incongruent trials that do not occur for congruent trials, and this additional main effect of previous congruency type may obscure what happens precisely in the interaction between the previous and the current trial. Still, the debate on which exact cognitive control mechanism is at play in the CSE may be the next relevant question.

Finally, the results leave room for further theoretical speculation. In particular, our observation of a CSE in the absence of feature repetitions and biased contingencies does not imply that the feature integration and contingency account should be rejected as partly or even fully accounting for CSE-like patterns when such effects are not ruled out a priori. Rather, as indicated above, this opens up the intriguing question of how these mechanisms interact and work together in producing adaptive behaviour. Further research should aim to systematically explore their respective influence on performance. The paradigms we have discussed in the present paper constitute an excellent starting point for such endeavour and may set a new standard for further examination of conflict adaptation –the *CSE 3.0*.

## Supporting Information

Dataset S1 **Data of the Stroop task in Experiment 1.**
(CSV)

Dataset S2 **Data of the picture-word interference task in Experiment 1.**
(CSV)

Dataset S3 **Data of the flanker task in Experiment 1.**
(CSV)

Dataset S4 **Data of the flanker task in Experiment 2A.**
(CSV)

Dataset S5 **Data of the flanker task in Experiment 2B.**
(CSV)

Table S1 **Stimuli used in the picture-word interference task of Experiment 1.** Sets of congruent and incongruent picture-word pairings used in the picture-word interference task of Experiment 1. Each set contains 24 unique congruent and incongruent picture-word pairings (English translation in italics). Participants were presented each of these five sets in random order.
(DOCX)
