# Supplementary material for: The Congruency Sequence Effect 3.0: A Critical Test of Conflict Adaptation
Source: PLoS One. 2014 Oct 23;9(10):e110462. doi: 10.1371/journal.pone.0110462 (PMC4207697; doi:10.1371/journal.pone.0110462)
Supplement: Table S1 — Stimuli used in the picture-word interference task of Experiment 1. Sets of congruent and incongruent picture-word pairings used in the picture-word interference task of Experiment 1. Each set contains 24 unique congruent and incongruent picture-word pairings (English translation in italics). Participants were presented each of these five sets in random order. (DOCX) [file pone.0110462.s006.docx]

| **Set 1** | | **Set 2** | | **Set 3** | | **Set 4** | | **Set 5** | | |
| --- | --- | --- | --- | --- | --- | --- | --- | --- | --- | --- |
| *Picture* | *Word* | *Picture* | *Word* | *Picture* | *Word* | *Picture* | *Word* | | *Picture* | *Word* |
| oog *eye* | oog *eye* | huis *house* | huis *house* | boek *book* | boek *book* | deur *door* | deur *door* | | tafel *table* | tafel *table* |
| voet *foot* | voet *foot* | auto *car* | auto *car* | hond *dog* | hond *dog* | stoel *chair* | stoel *chair* | | paard *horse* | paard *horse* |
| boom *tree* | boom *tree* | zak *bag* | zak *bag* | blad *leaf* | blad *leaf* | fles *bottle* | fles *bottle* | | bloem *flower* | bloem *flower* |
| ei *egg* | ei *egg* | schoen *shoe* | schoen *shoe* | bus *bus* | bus *bus* | ster *star* | ster *star* | | fiets *bike* | fiets *bike* |
| zon *sun* | zon *sun* | hoed *hat* | hoed *hat* | mes *knife* | mes *knife* | doos *box* | doos *box* | | kruis *cross* | kruis *cross* |
| bril *glasses* | bril *glasses* | ring *ring* | ring *ring* | vlag *flag* | vlag *flag* | slang *snake* | slang *snake* | | tent *tent* | tent *tent* |
| pijp *pipe* | pijp *pipe* | kaars *candle* | kaars *candle* | leeuw *lion* | leeuw *lion* | pijl *arrow* | pijl *arrow* | | vork *fork* | vork *fork* |
| worst *sausage* | worst *sausage* | peer *pear* | peer *pear* | spin *spider* | spin *spider* | schaar *scissors* | schaar *scissors* | | uil *owl* | uil *owl* |
| clown *clown* | clown *clown* | zwaan *swan* | zwaan *swan* | iglo *igloo* | iglo *igloo* | zaag *saw* | zaag *saw* | | hand *hand* | hand *hand* |
| bed *bed* | bed *bed* | kerk *church* | kerk *church* | glas *glass* | glas *glass* | oor *ear* | oor *ear* | | vis *fish* | vis *fish* |
| neus *nose* | neus *nose* | maan *moon* | maan *moon* | kat *cat* | kat *cat* | pan *pan* | pan *pan* | | koe *cow* | koe *cow* |
| lamp *lamp* | lamp *lamp* | kip *chicken* | kip *chicken* | wiel *wheel* | wiel *wheel* | beer *bear* | beer *bear* | | heks *witch* | heks *witch* |
| kroon *crown* | kroon *crown* | aap *ape* | aap *ape* | bom *bomb* | bom *bomb* | spook *ghost* | spook *ghost* | | kous *sock* | kous *sock* |
| vos *fox* | vos *fox* | kam *comb* | kam *comb* | bank *bench* | bank *bench* | slee *sled* | slee *sled* | | ui *onion* | ui *onion* |
| bh *bra* | bh *bra* | slak *snail* | slak *snail* | vuur *fire* | vuur *fire* | tand *tooth* | tand *tooth* | | brood *bread* | brood *bread* |
| tak *branch* | tak *branch* | berg *mountain* | berg *mountain* | kast *cupboard* | kast *cupboard* | bad *bath* | bad *bath* | | klok *clock* | klok *clock* |
| mand *basket* | mand *basket* | pop *doll* | pop *doll* | noot *nut* | noot *nut* | wolf *wolf* | wolf *wolf* | | piano *piano* | piano *piano* |
| vaas *vase* | vaas *vase* | geit *goat* | geit *goat* | taart *cake* | taart *cake* | schelp *shell* | schelp *shell* | | zweep *whip* | zweep *whip* |
| schop *shovel* | schop *shovel* | maïs *corn* | maïs *corn* | das *tie* | das *tie* | pomp *pump* | pomp *pump* | | strik *bow* | strik *bow* |
| kers *cherry* | kers *cherry* | lat *ruler* | lat *ruler* | tol *top* | tol *top* | krab *crab* | krab *crab* | | ton *barrel* | ton *barrel* |
| ijsje *ice cream* | ijsje *ice cream* | haai *shark* | haai *shark* | pizza *pizza* | pizza *pizza* | jojo *yoyo* | jojo *yoyo* | | tank *tank* | tank *tank* |
| boot *boat* | boot *boat* | wolk *cloud* | wolk *cloud* | hek *fence* | hek *fence* | eend *duck* | eend *duck* | | kraan *faucet* | kraan *faucet* |
| muis *mouse* | muis *mouse* | trui *sweater* | trui *sweater* | vogel *bird* | vogel *bird* | bijl *axe* | bijl *axe* | | hert *deer* | hert *deer* |
| tang *pliers* | tang *pliers* | naald *needle* | naald *needle* | rits *zipper* | rits *zipper* | kurk *cork* | kurk *cork* | | broek *pants* | broek *pants* |
| tafel *table* | deur *door* | oog *eye* | broek *pants* | huis *house* | oog *eye* | boek *book* | huis *house* | | deur *door* | boek *book* |
| paard *horse* | stoel *chair* | voet *foot* | tafel *table* | auto *auto* | voet *foot* | hond *dog* | auto *auto* | | stoel *chair* | hond *dog* |
| bloem *flower* | fles *bottle* | boom *tree* | paard *horse* | zak *bag* | boom *tree* | blad *leaf* | zak *bag* | | fles *bottle* | blad *leaf* |
| fiets *bike* | ster *star* | ei *egg* | bloem *flower* | schoen *shoe* | ei *egg* | bus *bus* | schoen *shoe* | | ster *star* | bus *bus* |
| kruis *cross* | doos *box* | zon *sun* | fiets *bike* | hoed *hat* | zon *sun* | mes *knife* | hoed *hat* | | doos *box* | mes *knife* |
| tent *tent* | slang *snake* | bril *glasses* | kruis *cross* | ring *ring* | bril *glasses* | vlag *flag* | ring *ring* | | slang *snake* | vlag *flag* |
| vork *fork* | pijl *arrow* | pijp *pipe* | tent *tent* | kaars *candle* | pijp *pipe* | leeuw *lion* | kaars *candle* | | pijl *arrow* | leeuw *lion* |
| uil *owl* | schaar *scissors* | worst *sausage* | vork *fork* | peer *pear* | worst *sausage* | spin *spider* | peer *pear* | | schaar *scissors* | spin *spider* |
| hand *hand* | zaag *saw* | clown *clown* | uil *owl* | zwaan *swan* | clown *clown* | iglo *igloo* | zwaan *swan* | | zaag *saw* | iglo *igloo* |
| vis *fish* | oor *ear* | bed *bed* | hand *hand* | kerk *church* | bed *bed* | glas *glass* | kerk *church* | | oor *ear* | glas *glass* |
| koe *cow* | pan *pan* | neus *nose* | vis *fish* | maan *moon* | neus *nose* | kat *cat* | maan *moon* | | pan *pan* | kat *cat* |
| heks *witch* | beer *bear* | lamp *lamp* | koe *cow* | kip *chicken* | lamp *lamp* | wiel *wheel* | kip *chicken* | | beer *bear* | wiel *wheel* |
| kous *sock* | spook *ghost* | kroon *crown* | heks *witch* | aap *monkey* | kroon *crown* | bom *bomb* | aap *monkey* | | spook *ghost* | bom *bomb* |
| ui *onion* | slee *sled* | vos *fox* | kous *sock* | kam *comb* | vos *fox* | bank *bench* | kam *comb* | | slee *sled* | bank *bench* |
| brood *bread* | tand *tooth* | bh *bra* | ui *onion* | slak *snail* | bh *bra* | vuur *fire* | slak *snail* | | tand *tooth* | vuur *fire* |
| klok *clock* | bad *bathtub* | tak *branch* | brood *bread* | berg *mountain* | tak *branch* | kast *cupboard* | berg *mountain* | | bad *bath* | kast *cupboard* |
| piano *piano* | wolf *wolf* | mand *basket* | klok *clock* | pop *doll* | mand *basket* | noot *nut* | pop *doll* | | wolf *wolf* | noot *nut* |
| zweep *whip* | schelp *shell* | vaas *vase* | piano *piano* | geit *goat* | vaas *vase* | taart *cake* | geit *goat* | | schelp *shell* | taart *cake* |
| strik *bow* | pomp *pump* | schop *shovel* | zweep *whip* | maïs *corn* | schop *shovel* | das *tie* | maïs *corn* | | pomp *pump* | das *tie* |
| ton *barrel* | krab *crab* | kers *cherry* | strik *bow* | lat *ruler* | kers *cherry* | tol *tod* | lat *ruler* | | krab *crab* | tol *tod* |
| tank *tank* | jojo *yoyo* | ijsje *ice cream* | ton *barrel* | haai *shark* | ijsje *ice cream* | pizza *pizza* | haai *shark* | | jojo *yoyo* | pizza *pizza* |
| kraan *faucet* | eend *duck* | boot *boat* | tank *tank* | wolk *cloud* | boot *boat* | hek *fence* | wolk *cloud* | | eend *duck* | hek *fence* |
| hert *deer* | bijl *axe* | muis *mouse* | kraan *faucet* | trui *sweater* | muis *mouse* | vogel *bird* | trui *sweater* | | bijl *axe* | vogel *bird* |
| broek *pants* | kurk *cork* | tang *pliers* | hert *deer* | naald *needle* | tang *pliers* | rits *zipper* | naald *needle* | | kurk *cork* | rits *zipper* |
